# National Influenza Annual Report 2023–2024: A focus on influenza B and public health implications

**DOI:** 10.14745/ccdr.v50i11a03

**Published:** 2024-11-07

**Authors:** Myriam Ben Moussa, Andrea Nwosu, Kara Schmidt, Steven Buckrell, Abbas Rahal, Liza Lee, Amanda Shane, Nathalie Bastien

**Affiliations:** 1 Centre for Emerging Respiratory Infections and Pandemic Preparedness, Public Health Agency of Canada, Ottawa, ON; 2 National Microbiology Laboratory Branch, Public Health Agency of Canada, Winnipeg, MB

**Keywords:** influenza B, influenza B/Yamagata, influenza B/Victoria, epidemic, paediatric, Canada

## Abstract

The 2023–2024 influenza epidemic saw the return of typical late-season influenza B circulation. The epidemic was declared in week 45 (week ending November 11, 2023) due to the predominant circulation of influenza A(H1N1) and peaked in week 52 (week ending December 30, 2023); however, as influenza A circulation decreased, influenza B detections and the percentage of tests positive increased, reaching its peak in week 14 (week ending April 6, 2024). Influenza B/Victoria dominated this wave of activity, contributing to the ongoing discussion about the apparent disappearance of influenza B/Yamagata. With the recommendation for the removal of influenza B/Yamagata lineages from the recommended seasonal influenza vaccine components, the influenza surveillance community is preparing for the possibility of a new seasonal pattern dominated by influenza B/Victoria circulation. This season, as a result of influenza B/Victoria’s overwhelming predominance, younger age groups were primarily affected by the wave of influenza B activity. Over the course of the season, among all influenza B detections, 52% occurred in children aged 0–19 years. Among all influenza B-associated hospitalizations, 46.4% were in children aged 0–19 years, and the highest cumulative hospitalization rates for influenza B were among children younger than five years (n=37 per 100,000 population) and children between the ages of 5–19 years (n=15 per 100,000 population). Continued vigilance and surveillance around influenza B trends and epidemiology is required to contribute to effective epidemic preparedness.

## Introduction

The COVID-19 pandemic has reshaped the global landscape of influenza surveillance, disrupting long-standing seasonal trends while presenting public health authorities with new challenges. Following the 2020–2021 season, which saw no nationally declared influenza epidemic, the 2021–2022 season was marked by a late and brief spring epidemic, reflecting the unpredictable nature of viral patterns. The 2022–2023 season, against the backdrop of a concerning “tripledemic” ((1)), is when Canada experienced its first fall epidemic since the 2019–2020 season. Continuing this trend, the first half of the 2023–2024 season saw classic influenza A circulation, but was marked by the return of typical late-season influenza B circulation for the first time since the onset of the pandemic, highlighting persistent shifts in influenza dynamics. Influenza B circulation has been the subject of its own shifting landscape, predating the pandemic itself. In pre-pandemic seasons, influenza B lineage dominance often alternated from one season to another with no apparent pattern, switching between B/Victoria to B/Yamagata. The dominant influenza B strain in the 2023–2024 season was influenza B/Victoria, as it has been since the 2018–2019 season.

This surveillance report summarizes the trends observed during the 2023–2024 influenza season in Canada, with a focus on the trends and implications surrounding the return of influenza B/Victoria circulation. For a more fulsome analysis of the 2023–2024 season, please refer to the week 34 FluWatch report ((2)).

## Methods

FluWatch is Canada’s long-standing influenza surveillance system, which monitors the spread of influenza and influenza-like illness through core surveillance indicators based on global epidemiological standards. FluWatch is a composite surveillance system that consists of seven key areas: virological surveillance; geographic spread; syndromic surveillance; severe outcome surveillance; outbreak surveillance; influenza strain characterization; and vaccination monitoring. Annually, influenza surveillance is conducted across Canada from epidemiological week 35 to week 34 of the following year. For the 2023–2024 Canadian influenza season, this surveillance period began on August 27, 2023, and ended on August 24, 2024. Detailed methods, including surveillance indicator definitions, data sources and statistical analyses can be found on the Public Health Agency of Canada’s FluWatch website ((3)).

## Results

### Laboratory detections

**Figure 1:**
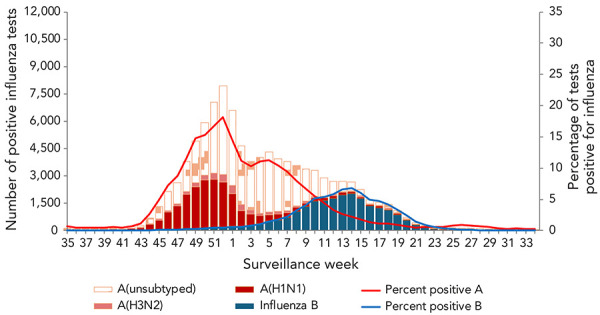
Number of positive influenza tests by type and subtype and percentage of tests positive, by report week, Canada, season 2023–2024

The 2023–2024 influenza epidemic in Canada began in week 45 (week ending November 11, 2023), when the national percentage of tests positive for influenza exceeded the 5% seasonal threshold (5.07%, 1,344 detections) ((4)). At the beginning of the epidemic, the majority of influenza detections were influenza A with influenza A(H1N1) being the dominant subtype. The epidemic peaked in week 52 (week ending December 30, 2023) at 18.7% of tests positive for influenza. At this point in the season, influenza A was still the dominant type detected. Following this eight-week period of increase (weeks 45–52), the percentage of tests positive for influenza A began to decrease, concurrently with the increase in the percentage of tests positive for influenza B in week 52. Influenza B reached its peak in week 14 (week ending April 6, 2024) at 6.8% tests positive and was the dominant type detected from weeks 11 to 22. This season, there were 103,173 influenza detections reported, of which 23% (n=23,233) were influenza B. These trends are summarized in **Figure 1**.

### Age distribution of detections

**Figure 2:**
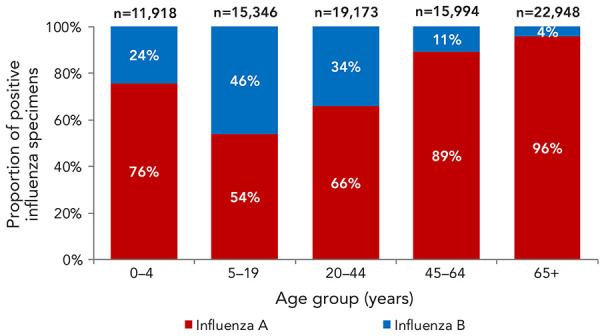
Proportion of positive influenza specimens by type and age-group reported through case-based laboratory reporting, Canada, season 2023–2024

During the 2023–2024 season, in keeping with historical trends, influenza B primarily affected younger age groups. Among all influenza infections in adults aged 65 years and older, only 4% were due to influenza B. Among those aged 45 to 64 years, only 11% of cases were due to influenza B; however, among younger age groups, a much higher proportion of total influenza detections were due to influenza B. For instance, among children aged 0–4 years, 5–19 years and 20–44 years, 24%, 46%, and 34% of detections, respectively, were due to influenza B (**Figure 2**).

**Figure 3:**
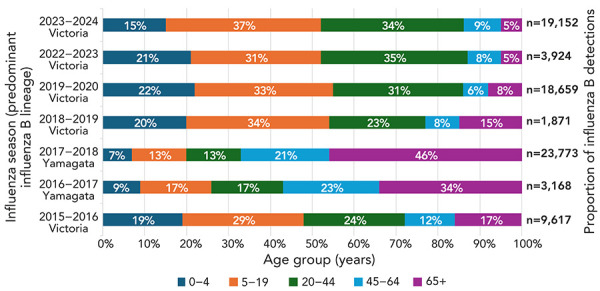
Proportion of influenza B detections by age group according to seasonal influenza B lineage dominance, Canada, seasons 2015–2016 to 2019–2020 and 2022–2023 to 2023–2024^a^ ^a^ The 2020–2021 and 2021–2022 seasons are excluded from this figure due to lack of substantial influenza circulation and the 2019–2020 season was truncated due to the COVID-19 pandemic

The 2023–2024 season was marked by the return of pre-pandemic influenza B circulation. **Figure 3** compares the age distribution of influenza B detections with accompanying age information from the current season back to the 2015–2016 season. Although in seasons 2015–2016 to 2019–2020, both B/Yamagata and B/Victoria circulated concurrently, for those seasons where B/Victoria was the dominant influenza B type, a greater proportion of detections were reported in individuals younger than 19 years (Figure 3). Conversely in seasons where B/Yamagata was the dominant influenza B type, the majority of detections occurred in older adults, aged 45 to 64 years and 65 years and older. From the seasons 2021–2022 onwards, there has been no detections of circulating B/Yamagata in Canada or abroad ((5)). Over the course of the 2023–2024 influenza B/Victoria season, among all influenza B detections, more than half (52%) occurred in children aged zero to 19 years. Influenza B/Yamagata and influenza B/Victoria both seem to affect those aged 20 to 44 years; however, this age group appears to be most affected by influenza B/Victoria.

### Strain characterizations

From September 1, 2023, to August 15, 2024, the National Microbiology Laboratory Branch has characterized 1,999 influenza viruses (334 A(H3N2), 920 A(H1N1) and 745 influenza B) received from Canadian laboratories.

Influenza B viruses can be divided into two antigenically distinct lineages represented by B/Yamagata/16/88 and B/Victoria/2/87 viruses. The recommended influenza B components for the 2023–2024 Northern Hemisphere influenza vaccine are B/Austria/1359417/2021 (Victoria lineage) and B/Phuket/3073/2013 (Yamagata lineage). All 745 influenza B viruses characterized were antigenically similar to B/Austria/1359417/2021. Additionally, 556 influenza B viruses underwent testing for antiviral resistance and all were sensitive to oseltamivir and zanamivir.

Age of detections by influenza B strain were provided by the National Microbiology Laboratory dating from the 2014–2015 season up to July 2024. Over a total of 6,070 detections for which age and influenza B strain information were provided, a clear trend emerged with respect to the age distribution of influenza B/Victoria detections and influenza B/Yamagata detections (**Table 1**). Influenza B/Victoria detections are concentrated among younger age groups, with nearly 60% of detections occurring in children aged 0–19 years; however, the trend is reversed in influenza B/Yamagata detections, with more than 50% of detections occurring in the two oldest age groups (45–64 years and 65 years and older).

**Table 1:** Influenza B viruses characterized by the National Microbiology Laboratory Branch by age group, seasons 2014–2015 to 2023–2024

Age group (years)	Influenza B viruses characterized by the National Microbiology Laboratory Branch (seasons 2014–2015 to 2023–2024)		
	Victoria (N=2,577)	Yamagata (N=3,493)	Total (N=6,070)
0–4	503 (19.5%)	258 (7.4%)	761 (12.5%)
5–19	994 (38.6%)	669 (19.2%)	1,663 (27.4%)
20–44	715 (27.7%)	566 (16.2%)	1,281 (21.1%)
45–64	208 (8.1%)	906 (25.9%)	1,114 (18.4%)
65 and older	157 (6.1%)	1,094 (31.3%)	1,251 (20.6%)

### Outbreaks

**Figure 4:**
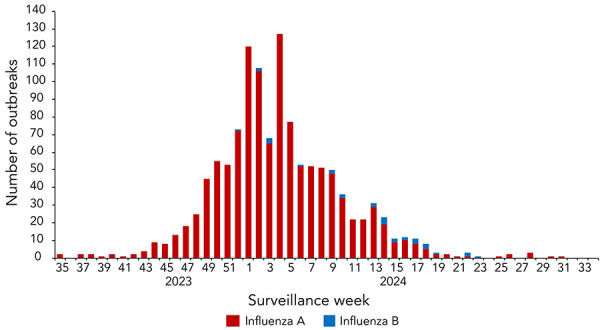
Number of laboratory-confirmed influenza-associated outbreaks by type, Canada, season 2023–2024

In the 2023–2024 season, a total of 1,224 influenza-associated outbreaks were reported. The majority (53%) were declared in long-term care facilities, followed by 27% in acute care facilities. Despite the wave of influenza B activity late in the season (starting in week 52, week ending December 20, 2023), outbreaks reported during this wave remained primarily associated with influenza A (**Figure 4**). In total, very few outbreaks this season were associated with influenza B, which is typical in pre-pandemic influenza seasons ((6)) (31 outbreaks or 2.5% of reported laboratory-confirmed outbreaks). Among long-term care facilities, 3% of reported laboratory-confirmed outbreaks were associated with influenza B.

### Severe outcomes

**Figure 5:**
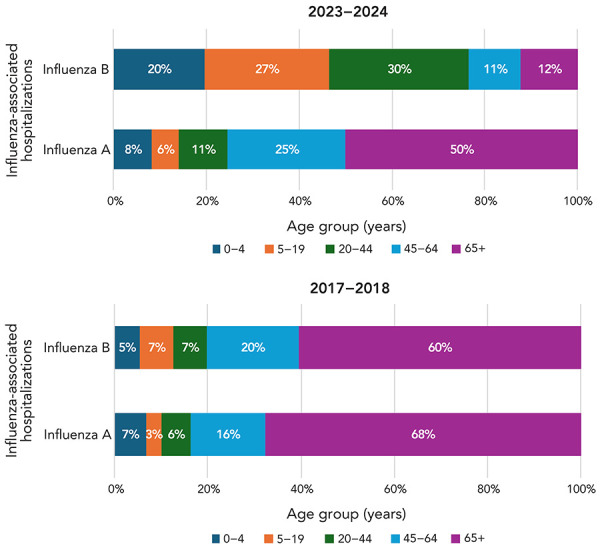
Influenza-associated hospitalizations by type and age group, Canada, season 2023–2024 compared to season 2017–2018

In the 2023–2024 season, 4,516 influenza-associated hospitalizations were reported by participating provinces and territories (influenza-associated hospitalizations are reported by Alberta, New Brunswick, Newfoundland and Labrador, Prince Edward Island and Yukon). Among these, 597 (13%) were due to influenza B. Among influenza B hospitalizations, 46.4% were among children aged 0–19 years, compared to only 12.2% among adults aged 65 years and older. This is in contrast to influenza A, where among the 3,919 influenza A hospitalizations, 14.1% occurred among children aged 0–19 years, compared to 50% among adults aged 65 years and older (**Figure 5**). These hospitalizations trends contrast the most recent influenza B/Yamagata dominant season in 2017–2018, where the age distribution of influenza-associated hospitalizations was similar between influenza A and influenza B (notably, the majority of hospitalizations, regardless of influenza type occurred in adults aged 65 years and older). These two seasons are the most comparable in terms of influenza B activity, as they are the most similar with respect to the total number of influenza B detections and only differ in dominant B lineage in circulation. In 2017–2018, a total of 23,772 influenza B detections were reported ((7)), compared to 23,233 detections in the 2023–2024 season.

**Figure 6:**
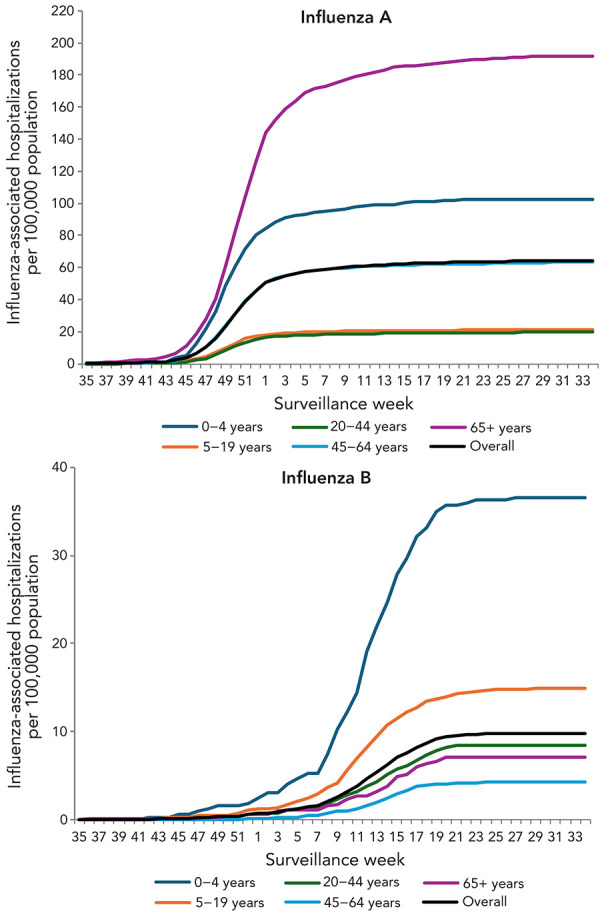
Cumulative rates of influenza-associated hospitalizations by age-group, type and surveillance week, Canada, participating provinces and territories^a,b^ ^a^ Note the different scales used in the two figure panels ^b^ These figures are likely an underestimate of the true cumulative hospitalization rates due to the small number of reporting provinces and territories over the course of the season

Nearly all influenza B-associated hospitalizations occurred during the influenza B wave late in the season. The cumulative influenza A-associated hospitalization rate for the 2023–2024 season follows a typical pattern, plateauing around the season peak; however, the cumulative influenza B-associated hospitalization rate only plateaus following the season’s influenza B peak (**Figure 6**). The highest cumulative hospitalization rates for influenza A were among adults aged 65 years and older (n=192 per 100,000 population) and children younger than five years (n=102 per 100,000 population), whereas the highest cumulative hospitalization rates for influenza B were among children younger than five years (n=37 per 100,000 population) and children between the ages of 5–19 years (n=15 per 100,000 population).

### Vaccine effectiveness

With contribution from the provinces of British Columbia, Alberta, Ontario and Québec, the Canadian Sentinel Practitioner Surveillance Network provides vaccine effectiveness estimates for the prevention of medically attended illness due to laboratory-confirmed influenza and COVID-19.

Between October 29, 2023, and May 4, 2024, influenza A(H1N1)pdm09 comprised about half, influenza B about one-quarter and influenza A(H3N2) about one-fifth of all influenza viruses detected by the Sentinel Practitioner Surveillance Network. All influenza B viruses were the vaccine matched B(Victoria) V1A.3a.2 clade. During the analysis period, vaccine effectiveness against any medically attended influenza was 46% (95% confidence interval [CI]: 37%–54%). Vaccine effectiveness against influenza B was 63% (95% CI: 48%–74%) ((8)).

For more information regarding influenza vaccine effectiveness for the 2023–2024 season, including information regarding influenza A vaccine effectiveness, please consult the week 34 FluWatch report ((2)).

## Discussion

The 2023–2024 influenza epidemic, in contrast with the several prior extraordinary seasons, appeared to be remarkably familiar. The return of pre-pandemic expected levels of influenza B activity stands out as a key feature of this season, while the persisting absence of influenza B/Yamagata raises questions about the evolving burden of influenza B. The apparent disappearance of influenza B/Yamagata has previously been discussed as a phenomenon that may incur notable implications on population level impacts of influenza B ((1)). While the 2022–2023 season did see typical late-season influenza B activity, it was less pronounced compared to the 2023–2024 season. In the 2022–2023 season, influenza B percent positivity reached a peak of 1.9% ((9)), compared to this season’s peak of 6.8%. The return of pre-pandemic-scale influenza B activity has been observed across North America and other regions of the Northern Hemisphere ((10)); yet according to global sources, no naturally occurring detection of influenza B/Yamagata has been confirmed worldwide since March 2020 ((4)). Since March 2020, only sporadic detections of influenza B/Yamagata have been reported, primarily from vaccine-derived cases or attributed to data entry errors ((11,12)).

The global shift towards influenza B/Victoria predominance has several implications for the field of seasonal influenza surveillance. Notably, the marked difference in affected age groups during B/Victoria seasons compared with B/Yamagata seasons implies a shift in burden of influenza B from older to younger age groups ((11,13)). It is important to note that influenza B typically circulates in much lower volumes than influenza A ((1)). While influenza A is the main contributor to the global burden of influenza, influenza B trends can push or pull seasonal severity by impacting the demographics upon which the burden falls.

Influenza B/Victoria is associated to a greater rate of infection in children ((14)). This demographic trend is apparent when comparing the age distribution of influenza B detections from season to season. Analysis of three influenza B/Victoria-dominant seasons (2023–2024, 2018–2019 and 2015–2016) indicates that detections occur primarily in younger age groups (0–19 years), as well as in those aged 20–44 years. The opposite is true of the B/Yamagata-dominant seasons illustrated (2017–2018 and 2016–2017), where older age groups (45 years and older) account for over 50% of seasons influenza B detections. This is further demonstrated through the age-specific strain characterization data for influenza B detections from the National Microbiology Laboratory Branch. Although not all detections are characterized every season, the random sample of influenza B detections that have been characterized, notably in seasons where influenza B/Victoria and influenza B/Yamagata co-circulated, reflect the consistent lineage-based age distribution.

Hospitalization data from this season and seasons past provides valuable insight into the impact of influenza B/Victoria on young children and youths. When comparing the age distribution of influenza-associated hospitalizations by age group between a B/Victoria-dominant season and a B/Yamagata-dominant season it is evident that there is a shift in the distribution of hospitalizations among age groups. While pediatric influenza B-associated hospitalizations account for nearly half of all influenza B associated hospitalizations in the 2023–2024 B/Victoria season, they only account for 12% of all influenza B associated hospitalizations in the 2017–2018 B/Yamagata season. There is a perception that influenza B infections are milder than those of influenza A; however, there are emerging studies that suggest variation in severity among the pediatric population, especially the hospitalized pediatric population ((15)). This underscores the importance of seasonal influenza vaccination among the pediatric population. Seasonal influenza vaccination remains a crucial component of seasonal influenza prevention practices. This season, influenza vaccination effectiveness against medically attended influenza B was 63% (95% CI: 48%–74%).

This shift in burden may also be perceived as a reduction in the burden of influenza on older demographics. Pre-pandemic, older demographics were consistently exposed to a higher risk of severe outcomes from influenza infection throughout the season. However, a shift in the burden of influenza B towards younger demographics as a result of the predominance of influenza B/Victoria may result in a reduction in overall disease burden relative to pre-pandemic years ((11,16)). This is evidenced by both the influenza type-specific outbreaks trends and the cumulative hospitalization rates for the 2023–2024 season. Despite the distinct wave of influenza B activity in the second half of the season, influenza B associated outbreaks in long-term care facilities, where the majority of residents are older adults aged 65 years and older, accounted for 3% of reported outbreaks. Among the hospitalization data, the highest cumulative influenza A-associated hospitalization rates were reported in adults aged 65 years and older (n=192 per 100,000 population) followed by those aged 0–4 years (n=102 per 100,000 population). However, the difference in magnitude between these two demographics is more pronounced when analyzing the influenza B associated cumulative hospitalization rate, where children aged 0–4 years face the highest burden (n=37 per 100,000 population)—more than five times higher than that of adults aged 65 years and older (n=7 per 100,000 population). These figures are likely an underestimate of the true cumulative hospitalization rates due to the small number of reporting provinces and territories over the course of the season. Nonetheless, the trends remain apparent, and older demographics have been less affected by influenza B/Victoria-dominant seasons. Continued monitoring for changes to the burden of influenza B/Victoria will be important to understand whether influenza B/Victoria trends are evolving, and how these trends are affecting the overall burden of influenza.

Despite the lack of circulation of naturally occurring influenza B/Yamagata, the extinction of the lineage has not yet been declared, nor has there been any scientific expert discussion on the criteria required to declare its extinction ((12)). The continued characterization of influenza B specimens is required to monitor for the potential resurgence of B/Yamagata. With the global move of surveillance programs to engage in the surveillance of multiple priority respiratory pathogens (SARS-CoV-2, influenza and respiratory syncytial virus) using one integrated surveillance platform, it is important to consider that a “one-size-fits-all” approach may not be sufficient to monitor the nuances of the influenza virus. The evolving landscape of influenza viruses is marking the beginning of a precarious phase for respiratory virus surveillance: future seasonal vaccines will no longer include the influenza B/Yamagata strain, new respiratory syncytial virus therapeutics have the potential to alter viral circulation dynamics in certain populations, and SARS-CoV-2 continues to co-circulate at various phases of the season. To effectively inform influenza-specific prevention policies, it is essential to maintain all components, both virological and epidemiological, of comprehensive surveillance programs such as FluWatch.
